# Using personal narrative to promote person-centered values in aging, dementia, and caregiving

**DOI:** 10.3389/fneur.2023.1167895

**Published:** 2023-09-18

**Authors:** Jennifer Merrilees, Cliff Mayotte, Erin Vong, Mindy Matice, Caroline Prioleau

**Affiliations:** ^1^University of California San Francisco Memory and Aging Center and Global Brain Health Institute, San Francisco, CA, United States; ^2^Voice of Witness, San Francisco, CA, United States

**Keywords:** oral history, personal narrative, values, aging, dementia oral history, dementia

## Abstract

Personal narrative is a powerful way to include people in their care and to understand their values that drive their needs. In this paper, we describe a program designed to teach oral history to clinicians and trainees in the field of aging, dementia and caregiving. The training uses empathic listening, open-ended interviewing, and the discovery of individual values and experience to breakdown stigma and preconceptions of what it means to age with cognitive impairment. Sharing these stories of aging, dementia, and caregiving becomes an important tool to break down stereotypes, promote person-centered care, and advocate for the unheard. The profound impact of the oral history process is felt by the narrator, the interviewer and the listener. Human beings are wired for stories, and oral history taps into that power to connect us and provide better care through better understanding.

## Introduction

Personal stories are a powerful way to share the rich, multi-dimensional nature of people’s experiences with aging, dementia, and caregiving. Personal experience offers a bridge to building connection and empathy while breaking down stereotypes and stigma. The Oral History Association defines oral history as the process of eliciting personal narrative by listening to and sharing stories.^1^ It involves active listening and the use of open-ended questions that foster sharing of the thoughts, feelings, experiences and values of the narrator. Narrative and personal stories hold a vital place in promoting social justice. The late historian Howard Zinn (1) popularized the practice of “people’s history,” which creates space for underrepresented accounts from individuals whose stories complicate or contradict dominant narratives. Oral history seeks to turn the microphone from the constantly amplified voices of the powerful and direct it toward ordinary people with stories that need to be heard. These stories become powerful testimonies that help us make sense of our world (2).

In health care, this process is often referred to as narrative medicine with the aim of promoting a deep understanding of the individual values and context that guide a patient’s and family’s decision-making while fostering a sense of dignity (3, 4). Personal narratives are representative of an individual’s experience in life and may be used to relieve psychosocial and existential distress (5). Narrative medicine techniques enhance the collaboration, empathy, and trust between a provider and the patient and help to reduce the perception of a power dynamic (6, 7). While many healthcare providers believe that open-ended interviewing and narrative techniques slow down assessment, almost 80% of patients were able to convey their initial concerns within two minutes when the provider engaged in active listening (8). Telling a personal narrative can be a mechanism for discovering meaning and purpose through a re-evaluation of important life events (5). Personal narratives also have the power to break down stereotypes and othering generalizations that can lump people together into broad categories at the expense of a person’s humanity.

Using the oral history process to promote social justice in the arena of aging, dementia, and caregiving brought together two organizations, Voice of Witness (VOW) and the University of California San Francisco Memory and Aging Center (UCSF MAC). VOW fosters an empathy-based understanding of the human experience through an oral history approach. This nonprofit organization promotes social justice and human rights with programs designed to collect stories and amplify the voices of those impacted by, and fighting against, injustice. The mission of the MAC is to provide the highest quality of care for those with cognitive problems and to educate patients, families, the public, and healthcare professionals. The MAC runs the Hellman Visiting Artist Program that brings artists to learn about neurogenerative disorders and foster creative exchange between the artists, clinicians, people living with cognitive impairment, families, and the community. Artists participate for one year and during that time engage in educational opportunities, observe clinical and research encounters, and develop an artistic offering inspired and informed by their experience. The artist presents their creative output with the larger community through a public performance thus raising important awareness about the lived experience of dementia and dementia care. To date, Hellman artists have represented the fields of poetry, dance, music, photography, podcasting, filmmaking, playwriting, comedy, and sculpture.

Out of this shared interest in promoting social justice, VOW became the sixth Hellman Visiting Artist at the MAC. During this residency, VOW staff trained interested MAC staff (physicians, nurses, social workers, research assistants, scientists, and MAC alumni) on conducting oral history interviews, transcription, and the development of a completed “story.” Thirty stories were collected, and this launched a permanent and enduring relationship between VOW and the MAC that was named *hear/say*. During the next two years, *hear/say* was incorporated into the annual curriculum of the Atlantic Fellows for Equity in Brain Health at the Global Brain Health Institute (GBHI) due to the shared values in promoting empathy, raising awareness around issues of brain health, and serving vulnerable and underserved populations.

The purpose of this paper is to describe the *hear/say* project that spans several years and leverages a collaboration between academia, health care, and institutions with social justice values. We describe the elements of oral history training, process, and program participants. We share our outcomes relating to the objectives of promoting empathetic listening, open-ended interviewing, discovering individual values and experience, and sharing stories about aging, dementia, and caregiving. We discuss the value and our methodology in forging community partnerships.

## Materials and methods

The *hear/say* project team trains participants using an ethics-driven oral history framework developed by VOW (9) and modified to match the needs and experiences of the Atlantic Fellows program. The course gives participants an opportunity to experience every step of the oral history process, including ethics and best practices for building trust between narrators and interviewers, interviewing techniques, recording, transcribing, editing, storytelling, and sharing the stories publicly. The course culminates in an optional public reading (called Readers’ Theater) of excerpts from the collected stories, and the stories are added to the ongoing *hear/say* oral history archive.

### Participants

In its first year, the *hear/say* participants included physicians, nurses, social workers, research assistants, scientists, and alumni from the MAC. In subsequent years, the participants included the Atlantic Fellows for Equity in Brain Health program at the Global Brain Health Institute (GBHI) based at UCSF MAC and at Trinity College Dublin, the University of Dublin, Ireland. The fellows are a group of interprofessional experts in brain health, aging, and dementia who come from countries around the world and work together to promote brain health and dementia prevention, reduce stigma, and improve the quality of life of people with dementia. Fellows selected for the program represent a wide variety of disciplines including artists, researchers, public health specialists, journalists, health care providers, educators, administrators, and social scientists. Fellowships start in September and last for one year in residence with career-long accompaniment. Components of the fellowships include didactics on a range of topics, (e.g., clinical, ethical, health policy, and leadership) about aging and dementia and leadership development. Each fellow is mentored and asked to develop a project of regional interest that builds on their professional goals and knowledge about dementia. In the fall, the new fellows are invited to participate in an introductory *hear/say* oral history training with a subsequent elective course in the summer. Faculty and staff from GBHI and the MAC are also invited to participate in the course.

### Training

The *hear/say* project started outside of the regular GBHI curriculum but in its second year, became a part of the standard educational offerings for the fellows as a summer elective course. Fellows sign up for the elective hear/say course, which consists of a series of workshops to teach the fundamentals of oral history and provide a guided experience through all the steps of the process. The training embodies the collaboration between GBHI and VOW and supplements the goal-oriented interviews (for example, clinical histories) typically employed in clinical settings with training in open-ended, narrator-driven interviews that explore stories and topics meaningful to the narrator. We discuss that the use of the term “narrator” rather than interviewee is deliberate and fosters acknowledgment that the people interviewed have agency and choice in how they relate their story. The course is led by four co-authors and lead trainers (CM, JM, CP, and EV) with administrative, educational, and technical support from co-author MM. The learning objectives for the course are to increase fellows’ capacity for empathy, active listening, and authenticity and to create opportunities for fellows to further their practices and projects by utilizing personal narrative as a tool for positive social change.

Workshops were scheduled at the two founding sites in San Francisco, California and Dublin, Ireland. In the 2018 and 2019 academic years, 10 h of training were provided in person over the course of 5 days at each site. In 2020 and 2021, the training was done in four virtual sessions, each lasting 90 min (6 h total) with participants attending remotely from their home countries.

The workshops begin with an introduction to oral history and the empathy-based and ethics-driven storytelling methodology as practiced by VOW (9). This methodology emphasizes centering the narrator experience, active listening by the interviewer, consent, representation, community relationships, embodying empathy, and learning from first-person narratives. Participants read oral history excerpts from the VOW book series, including the *hear/say* books, and view short video clips from past Readers’ Theater events. This contextualizes the oral history techniques and provides participants with a basic road map for their own interviews during the course. The first workshop also includes a former narrator from the *hear/say* series, giving participants the chance to hear from and ask questions of someone who experienced the interview process firsthand.

The second workshop focuses on preparing interviewees for their oral history interviews. This preparation includes practice in crafting open-ended, story-generating questions, and how to create a safe and brave space for the sharing of stories. Participants also engage in practice interviews, taking turns asking each other about a personal artifact or object, to give each person an opportunity to experience the interview process as both narrator and interviewer. Sensitive issues can arise during the interview that can be emotionally distressful for both the narrator and the interviewer. As part of the training, participants are encouraged to share strategies in how to monitor for, and manage, emotional distress (9). The consent process is reviewed (explained in more detail below). The second workshop concludes reviewing the myriad logistics of recording and transcribing interviews in person and virtually. If participants cannot use their smartphone or videoconference software for recording, we provided audio recorders for them. We contracted with an automated transcription service, and we cover how to access and use this service. Participants are given resources to continue their learning by reading full transcripts of official interviews and narratives presented in other mediums, such as animated videos or graphic novels.^2^^,^^3^ In between the second and third workshops, participants conduct interviews with their chosen narrator(s). If participants do not have access to a narrator, VOW and MAC staff facilitate a connection with one.

The third workshop is an introduction to editing an oral history transcript using the interviews conducted. This process includes acknowledging that people speak differently than they read and write, and that much of the editing process focuses on shaping a narrative that would be clear to a reader yet respects and reflects the voice of the narrator. The VOW methodology takes a literary approach to craft a cohesive and fluid story out of an interview transcript while maintaining the narrator’s original words and intent. Narrators are given the opportunity to read their story before publication and consent to sharing or making changes as needed. Participants practice editing in small groups during the workshop to compare different editing choices. Each person gains valuable experience and insight into shaping a story with a clear narrative arc, while honoring the narrator’s agency and voice in presenting their story. Table 1 provides an example of story editing from an interview with a family caregiver of a person with dementia: the final edited version appeared in the first *hear/say* book (10).

**Table 1:** Example of story editing.

**Transcript excerpt (unedited):**
Interviewer (I): taking over some of those tasks?
Narrator (N): yeah, the day to day and such a gradual thing that you do not even notice it. You know it’s years before you realize oh, now I’m doing this and oh, now I’m doing this and what is he doing?
I: So, it really creeps up on you?
N: It really creeps up on you. You do not really even notice it because it’s just one more thing and you know, as a mother there’s always a gazillion things you are doing anyway and you are working and you are just trying to keep everything together and so, if Jim cannot do the dishes now, which was the one thing he loved to do, well then I guess I’ll do that too ummm it was yeah, it was so gradual and I think I’ve told you this before it was like a fog rolling in and rolling in and rolling in and you do not notice it. It just gets thicker and thicker and thicker and then it’s so thick and it’s been there for so long that you cannot remember what it was like when the sun was out. And you cannot remember what your life was like when you did not have to do everything ‘cause you just gradually take it on. Like a sponge I guess. You gradually absorb all the water.
I: was there ever a time that you thought you could not do it?
N: no, I do not think so. I mean, I was always such a competitive person. I mean, and stubborn, so it wasn’t an option to not do it. I mean I did try to divorce him (laughs) because his behavior was so bizarre and it just was not, he wasn’t living life the way I had imagined we would be living our life and he was pretty unrealistic and unreasonable.
**Final (edited) version:**
You do not really even notice becoming a caregiver because it’s just one more thing. And, as a mother there’s always a gazillion things you are doing anyway, and you are working, and you are just trying to keep everything together. And so, if Jim cannot do the dishes now, well then, I guess I’ll do that too. It was so gradual, and it was like a fog rolling in and rolling in and rolling in and you do not notice it. It just gets thicker and thicker and thicker and then it’s so thick and it’s been there for so long that you cannot remember what it was like when the sun was out. And you cannot remember what your life was like when you did not have to do everything, because you just gradually take it on. Like a sponge, I guess. You gradually absorb all the water. And yet, there was never a time that I thought, “I cannot do this.” I mean, I was always such a competitive person. And stubborn, so it wasn’t an option to not do it.

The final workshop is devoted to sharing brief excerpts of the in-progress oral history narratives. Participants create a “story circle” and celebrate their narrators by bringing their voices into a shared space by reading excerpts aloud. Even in brief excerpts, participants can share moments of joy, pain, struggle, heart, and humor. During this final workshop, the oral history process comes full circle by hearing from a former hear/say interviewer about how the process has impacted their work beyond the training and how lessons learned around narrator agency, empathy, representation, and active listening have been taken into real-world applications. The session concludes with planning and logistics for the public Readers’ Theater event at the end of the course. Before this final event, participants spend more time editing their oral history narratives with guidance from the hear/say team.

### Consent

Although Institutional Review Board approval and participant consent are not required for oral history work, all narrators in this project sign a consent form before participating. The form is a “rolling consent” that the hear/say team developed with input from the Atlantic Fellows and other interviewers. It centers on narrator agency and allows the narrator to select a level of sharing they are comfortable with. For example, some narrators may choose to not share their story outside of the interview or they may prefer to not use their full name, and the consent form allows them to make these choices. The consent form allows the narrator to choose how their story can be shared: in a published book, read aloud, and/or filmed, and it allows for the narrator to modify or withdraw their consent at any time during the process. The consent form has been translated into languages used by the *hear/say* participants and has, so far, been translated into traditional and simplified Chinese, French, Portuguese, Romanian, and Spanish.

### Ongoing consultation

The *hear/say* team provides follow-up and consultation for participant interviewers on all aspects of the experience including preparation of transcripts and stories, material for the Readers’ Theater event, and strategies for incorporating their interview skills and what they had learned from their narrators into project development and future work. Throughout the entire course, the hear/say team is in contact with the participants and hosts drop-in sessions in which the interviewers can show up to ask questions and clarify concepts.

## Results

During the first two years of *hear/say*, 13 interviewers that included MAC staff (former and current), Atlantic Fellows, and GBHI faculty and staff collected stories from 31 narrators from five different countries. Four translators and 16 story editors, including the 4 lead trainers, helped finalize the stories. The Readers’ Theater event was presented live to about 75 people and followed by a reception. Audience members included interviewers, narrators, colleagues, and the local community. In the third year, training was conducted remotely with 15 fellows and six staff participating. This resulted in ten interviewers who shared eight stories in four languages from five countries during a Readers’ Theater with an audience of 80 people. In the fourth year, training was conducted remotely with 9 fellows and six staff participating. This resulted in ten interviewers who shared ten stories in two languages from seven countries during a Readers’ Theater with an audience of 106 people. For all the Readers’ Theaters, we had the participation of additional readers who read excerpts of the multilingual stories (stories were shared in the narrator’s language first, followed by an English translation). During all four years, narrators included people living with cognitive impairment, caregivers, physicians, nurses, scientists, research staff, and artists. Two books have been published (10, 11) and four public Readers’ Theater events have been held. Videos of the readings can be seen at memory.ucsf.edu/caregiving-support/caregiver-well-being/hearsay and www.gbhi.org/projects/hearsay.

We include an excerpt from an interview with a man who had been diagnosed with Alzheimer’s disease. He participated in our first year of *hear/say* and a portion of his story was read at the Reader’s Theater [the complete transcribed story can be found in Prioleau, C. and Merrilees, J., ed. (10)].

Short of a solving the disease, the best way to help is by teaching people we aren’t vegetables yet. Just spreading the word. Because for many people, they think, “Oh my God, it’s the end of the world.” And it’s not the end of the world. It’s not great, but it’s not the end of the world. And that’s important to let people know. That’s why having our group is good, being able to teach people who are in the medical profession, and I think advocacy is great… people with the disease talking, telling our story. Sharing our story is so powerful, with any disease. If research needs to be done, we can tell them why by showing our story. And people should also know that you can still have a sense of humor when you have a disease. You live a certain amount of time, and then you plotz. And you do what you can do. I like being a teacher. So, I hope it stays in your head, because I’ll probably forget it!

## Discussion

The *hear/say* project, by teaching oral history methodology, is a valuable tool for clinical providers, researchers, teachers, and students/trainees in promoting an understanding of an individual’s values. Personal stories help to shed light on not only the experience of an individual but can help to forge changes in social policy that impact countless others. Oral history training has helped our staff and trainees to develop tools for building equity and promoting social justice in their work by shedding light on the rarely heard experiences.

In the *hear/say* training programs to date, interviewers and narrators expressed appreciation and gratitude for the opportunity to share their stories, to be heard, and to contribute to knowledge sharing about their experiences. When stories are told and listened to, they become the experience of both the narrator and the interviewer (12). The *hear/say* participants reported this multidirectional benefit: a deeper empathy and mutuality developed as they learned more about their narrators (13). Many reported that the process of conducting an oral history interview gave them new skills in communication and the art and benefits of listening. People have a strong desire to preserve their stories (2) and giving them a safe space to speak their stories is a critical step in forging mutual respect and understanding.

In our situation, exploring shared values among our community partners was a critical bridge to building and sustaining the *hear/say* project. In the first meetings between VOW and the MAC, the four trainers found common ground discussing the potential for blending our areas of expertise: for VOW this was vast experience in the oral history process and for the MAC having access to cohorts whose voices were often not heard or appreciated. We realized that we spoke the same language regarding our shared values of empathy, social justice, and reducing stigma and that each organization brought expertise that the other could grow from. Themes that have been associated with building strong community partnerships include but are not limited to the creation and nurturing of trust, respect for knowledge, community-defined goals, flexibility, compromise, capacity building, and attention to sustainability (14, 15). Values that promote partnerships include respect, communication, mutual benefit, and shared ownership with potential threats including power imbalances, lack of shared vision, and lack of time (16). Throughout the years of collaboration between VOW and GBHI, we continue to nurture these positive values and work on reducing potential threats.

## Summary

Aging, threats to brain health, and caregiving can be frightening, isolating, and stigmatizing experiences. Persons living with dementia and their caregivers report that their healthcare providers do not always show respect and knowledge about their culture and their values that drive their decisions (17). Healthcare providers, artists, scientists, and social policy change agents face enormous barriers and challenges in effecting change in this arena and need the tools and strategies necessary to inform change. Personal stories become a strategy for eliciting attention and understanding to these threats to health. The techniques of oral history— interviewing and honoring the agency and primacy of the narrator—are powerful and accessible strategies for understanding the experience and values of an individual whose story can then impact and inform others. In addition, a systematization of the themes within the personal narratives could provide an even more explicit bridge between the values of narrators and the promotion of social justice and healthcare change.

## Author contributions

CM, JM, EV, MM, and CP contributed to development of this paper, including the background, results, and discussion. CM and EV wrote the description of the training program. JM supervised all phases of the paper. All authors contributed to the article and approved the submitted version.

## Funding

Funding was provided by the Hellman Foundation and the Global Brain Health Institute.

## Conflict of interest

The authors declare that the research was conducted in the absence of any commercial or financial relationships that could be construed as a potential conflict of interest.

## Publisher’s note

All claims expressed in this article are solely those of the authors and do not necessarily represent those of their affiliated organizations, or those of the publisher, the editors and the reviewers. Any product that may be evaluated in this article, or claim that may be made by its manufacturer, is not guaranteed or endorsed by the publisher.

## Footnotes

^1^ https://www.oralhistory.org/about/do-oral-history

^2^ https://storycorps.org/animation/

^3^ https://brianfies.blogspot.com/2017/10/a-fire-story-complete.html
